# American Heart Month — February 2019

**DOI:** 10.15585/mmwr.mm6805a1

**Published:** 2019-02-08

Heart disease is the leading cause of death for men and women in the United States, and heart attacks are a major category of heart disease; someone in the United States has a heart attack every 40 seconds (*1*). February is American Heart Month, an ideal time to remind all adults to focus on their hearts and encourage them, their families, friends, and communities to learn the important signs and symptoms of heart attack and how to respond. Recognizing that someone might be having a heart attack and calling emergency services (9-1-1) are crucial for optimizing access to lifesaving emergency cardiac care and receipt of advanced treatments and improving survival. Five common symptoms of a heart attack are 1) pain or discomfort in the jaw, neck, or back; 2) feeling weak, lightheaded, or faint; 3) chest pain or discomfort; 4) pain or discomfort in the arms or shoulder; and 5) shortness of breath. If someone is suspected to be having a heart attack, 9-1-1 should be called immediately.

A report in this issue of *MMWR* shows that, although the percentage of persons who are aware of all five heart attack symptoms increased from 39.6% in 2008 to 50.2% in 2017, sociodemographic disparities existed (*2*). Education is needed to more widely disseminate information about how to recognize a possible heart attack and contact lifesaving emergency services.
